# Lamiaceae plants improve serum cholesterol, triglyceride, and LDL in patients with metabolic syndrome: A systematic review and meta‐analysis of randomized clinical trials

**DOI:** 10.1002/fsn3.4451

**Published:** 2024-09-15

**Authors:** Hossein Hassanpour, Marzieh Mojtahed, Aziz A. Fallah, Tina Jafari

**Affiliations:** ^1^ Department of Basic Sciences, Faculty of Veterinary Medicine Shahrekord University Shahrekord Iran; ^2^ Department of Cellular and Molecular Biology, Faculty of Advanced Sciences and Technology, Tehran Medical Sciences Islamic Azad University Tehran Iran; ^3^ Department of Food Hygiene and Quality Control, Faculty of Veterinary Medicine Shahrekord University Shahrekord Iran; ^4^ Department of Biochemistry and Nutrition, Faculty of Medicine Shahrekord University of Medical Sciences Shahrekord Iran

**Keywords:** cholesterol, HDL, Lamiaceae, LDL, lipid profile, meta‐analysis, triglyceride

## Abstract

The Lamiaceae plant family has a positive impact on metabolic diseases. This study meta‐analyzed the data of different clinical trial studies on the impacts of these plants on blood lipid biomarkers including cholesterol, triglyceride, high‐density lipoprotein (HDL), and low‐density lipoprotein (LDL). A comprehensive search was conducted using PubMed, Scopus, ISI Web of Science, ProQuest, Scientific Information Database, MagIran, and Google Scholar to identify relevant published research up to December 20, 2023. The meta‐analysis revealed that the Lamiaceae family significantly reduced cholesterol (*p* < .001), triglyceride (*p* = .003), and LDL (*p* < .001) levels. The rise in HDL levels was not significantly impacted (*p* = .069). Subgroup studies revealed that Lamiaceae plants improve all lipid parameters for a short duration (≤8 weeks) while in a longer duration (>8 weeks) only LDL level is improved in the blood (*p* < .05). In addition, Lamiaceae plants specifically decreased LDL and cholesterol levels in diabetic patients and only LDL levels in the individuals who were hyperlipidemic or overweight/obese. Within the Lamiaceae family, the genera *Satureja* and *Coleus* had the most significant effects in decreasing triglyceride and LDL/cholesterol levels, respectively. *Origanum* was the only genus within the Lamiaceae family that significantly improved three lipid parameters (cholesterol, LDL, and HDL). The meta‐analysis confirmed that Lamiaceae plants could improve the levels of lipid parameters (triglyceride, cholesterol, and LDL) in patients with metabolic syndrome.

## Graphical abstract

Lamiaceae family had a significant effect in reducing cholesterol, triglyceride, and LDL. Lamiaceae family had no significant effect in increasing HDL. Lamiaceae plants improve all lipid parameters for a short duration (≤8 weeks). Lamiaceae plants decreased LDL and cholesterol levels in diabetic patients and only LDL hyperlipidemic or overweight/obese individuals.

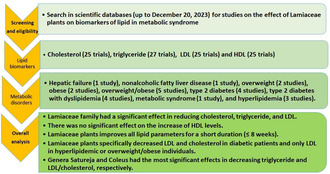

## 1 INTRODUCTION

Metabolic syndrome, which is characterized by overweight/obesity, diabetes, nonalcoholic fatty liver disease, and hyperlipidemia, is a rising public health issue despite significant advancements in the identification and formulation of innovative treatments (Guerra et al., 2021; Han & Lean, 2015). Medicinal herbs have long been recognized as significant sources of phytotherapeutic and/or preventative substances, primarily because of their widespread availability and relatively low risk (David et al., 2015). The Lamiaceae is a prominent family of flowering plants, characterized by its unique features. It consists of 12 subfamilies, 16 tribes, 9 subtribes, 236 genera, and about 7000 species found all over the globe (Frezza et al., 2019). Lamiaceae members have a global distribution, with some species being restricted to certain regions. Typically, these species thrive in tropical and temperate climates found at altitudes ranging from sea level to 2500 m above sea level (Frezza, 2018). A substantial proportion of plants pertaining to the Lamiaceae family are aromatic and include a diverse range of plants with significant biological and medicinal applications (Trivellini et al., 2016).

Plants pertaining to the Lamiaceae family have extensive use as food herbs and spices, including sage (*Salvia*), thyme (*Thymus*), mint (*Mentha*), oregano, marjoram (*Origanum*), rosemary (*Rosmarinus*), lavender (*Lavandula*), and basil (*Ocimum*). Additionally, these plants are utilized as flavor enhancers in the food industry (*Rosmarinus officinalis*, *Ocimum basilicum*, *Origanum majorana*), as perfumery constituents (mint and lavender), and in beverages and infusions (*Satureja montana*, *Mentha piperita*, *Salvia officinalis*, *Sideritis scardica*, etc.) (Agatonovic‐Kustrin et al., 2023; Stefanaki & van Andel, 2021). The therapeutic effects of the Lamiaceae species are ascribed to their abundant concentration of volatile and flavonoid chemicals (Trivellini et al., 2016). The many categories of phytochemical compounds found in Lamiaceae plant species, including phenolic acids, phenylpropanoids, monoterpenoids, diterpenoids, terpenoids, and flavonoids, along with their diverse biological functions, have been documented (Raja, 2012). Existing research indicates that Lamiaceae exhibits antioxidant, antibacterial, antiplatelet, antidiabetic, antihyperlipidemic, antiobesity, antisteatotic, hypercholesterolemic, anti‐inflammatory, and hepatoprotective effects (Bendif, 2021; Patrignani et al., 2021; Raja, 2012).

While there is an initial proof supporting the effectiveness of the Lamiaceae genus in treating metabolic‐related illnesses, the available research is still in its early stages and mostly lacks a comprehensive explanation of its specific mechanisms of action. Furthermore, the efficacy of these therapeutic herbal plants remains uncertain as a result of various constraints, necessitating the assessment of both preclinical and clinical investigations to validate the advantages of these plants. Lamiaceae plants have been the subject of several human clinical trials evaluating their potential therapeutic effects on metabolic illnesses. However, the findings have not yielded definitive conclusions. Hence, we performed a meta‐analysis to assess the effectiveness of consuming plants from the Lamiaceae family in influencing blood lipid biomarkers including cholesterol, triglyceride, high‐density lipoprotein (HDL), and low‐density lipoprotein (LDL) to get a greater insight into the subject.

## 2 MATERIALS AND METHODS

### 2.1 Strategy of searching

A comprehensive search was carried out using PubMed, Scopus, ISI Web of Science, ProQuest, Scientific Information Database (SID), MagIran, and Google Scholar (for gray literature) to identify relevant published research up to December 20, 2023. The following keywords were used in this search: (“lamiaceae*” [All Fields] OR “labiatae*” [All Fields] OR “basil” OR “rosemary” OR “mint*” OR “plectranthus*” OR “sage*” OR “tulsi*” OR “savory*” OR “marjoram*” OR “teucrium*” OR “vitex*” OR “oregano*” OR “hyssop*” OR “thyme*” OR “lavender*” OR “stachys*” OR “salba*” OR “perilla*” OR “salvia*” OR “chia*” OR “catnip*” OR “bee balm” OR “wild dagga” OR “oriental motherwort*” OR “scutellaria*” OR “ocimum*” OR “leucas*” OR “satureja*” OR “leonotis*” OR “zataria*” OR “coleus*” OR “origanum*” OR “rosmarinus*” OR “lavandula*” OR “ziziphora*” OR “basilicum*” OR “mentha*” OR “elsholtzia*” OR “micromeria*” OR “teucrium*” OR “thymbra*” OR “moluccella*” OR “dracocephalum*” OR “hymenocrater*” OR “thymus*” OR “marrubium*” OR “premna*” OR “pogostemon*” OR “mesona*” OR “orthosiphon*” OR “lycopus*” OR “melissa*” OR “lenotis*” OR “lycopus*”) AND (“cholesterol” OR “triglyceride” OR “nepeta*” OR “high‐density lipoprotein” OR “HDL” OR “hyptis*” OR “low‐density lipoprotein” OR “LDL” OR “lipoprotein” OR “VLDL” OR “lipid”) AND (“intervention study” [tiab] OR “intervention” [tiab] OR “controlled trial” [tiab] OR “randomized” [tiab] OR “randomised” [tiab] OR “random” [tiab] OR “randomly” [tiab] OR “placebo” [tiab] OR “assignment” [tiab] OR “clinical trial” [All Fields] OR “trial” [All Fields]) AND (“metabolic syndrome*” [tiab] OR “metabolic disorder*” [tiab] OR “metabolic*” [tiab] OR “diabetes*” [tiab] OR “hyperlipidemia*” [tiab] OR “dyslipidemia*” [All Fields] OR “overweight*” [All Fields] OR “obese*” [All Fields] OR “hypercholesterolemia*” [All Fields] OR “hypertriglyceridemia*” [All Fields]). The references of qualified studies and pertinent review articles (found through database searches) were examined manually in order to prevent losing publications.

### 2.2 Study eligibility

The subsequent criteria were utilized to choose the studies for this survey: (a) Randomized controlled clinical trials (RCTs) involving human subjects, employing parallel or crossover designs; (b) either provision of a placebo for the control group or differentiation between the control and intervention groups through the consumption of Lamiaceae; (c) provision of sufficient baseline and post‐intervention data on lipid profile biomarkers, including cholesterol, triglyceride, LDL, and HDL, for both groups.

### 2.3 Data extraction

The following details were extracted from articles: (a) trial parameters, such as the first author's last name, the publication date, the administered dosage and species of Lamiaceae family, the trial design and duration of follow‐up, the trial location, and the number of participants for each group; (b) metabolic status of participants; and (c) trial outcomes, such as cholesterol, triglyceride, LDL, and HDL levels. The Engauge Digitizer software version 12.1 was used in trials where the findings were shown graphically in order to acquire numerical data.

### 2.4 Evaluation of trial quality and overall outcomes

The Cochrane guideline was used to evaluate the potential for bias, using seven criteria, for every qualifying study. Trials were categorized into high, unclear, or low risk of bias for each criterion. Each trial was categorized based on its general quality as “Low,” “Fair,” or “Good” as previously described (Fallah et al., 2020). The NutriGrade rating system was utilized to assess the overall quality of each biomarker's summarized impact. According to Schwingshackl et al. (2016), this system consists of seven domains. Each biomarker is assigned a score ranging from 0 to 10, representing its overall impact. The scores ranging from 0 to 3.99, 4 to 5.99, 6 to 7.99, and 8 to 10 were categorized as very low, low, moderate, and high quality, respectively (Schwingshackl et al., 2016).

### 2.5 Data synthesis and analysis

The effect size for each trial was assessed using the unstandardized mean difference and a 95% confidence interval (95% CI). Using the subsequent formula, the net mean difference in the intervention and control groups was obtained: The difference between the measurement at the conclusion of the trial and the measurement at the baseline. The formula for calculating the standard deviation (SD) of the mean difference in the intervention and control groups is as follows: SD = square root of [(SD_baseline_)^2^ + (SD_end‐trial_)^2^ − (2R × SD_baseline_  × SD_end‐trial_)], assuming a correlation coefficient (R) of 0.50 (Jafari et al., 2019). For randomized controlled trials that provide the standard error of the mean (SEM), the standard deviation (SD) was determined using the following formula: SD = SEM × sqrt (*n*), where *n* is the total number of participants. A random effects meta‐analysis was conducted to ascertain the overall impact based on the computed effect sizes (Jafari et al., 2016). Subgroup analysis was conducted using parameters such as the genus of the Lamiaceae family, study durations, and metabolic status of participants. The Cochrane *Q*‐test was used to assess the variability between trials. The *I*‐squared (*I* ^2^) index was used to quantify the degree of heterogeneity across the trials. Heterogeneity values of 25%, 50%, and 75% were classified as low, moderate, and high heterogeneity, respectively (Jafari et al., 2015). A sensitivity analysis was conducted to assess the impact of a specific trial or number of trials on the overall effect, as well as to identify which trial(s) may be contributing to the heterogeneity (Askari et al., 2015; Fallah et al., 2018). Evaluation of publication bias was carried out using the tests of Egger's regression asymmetry and the Begg and Mazumdar adjusted rank correlation (Lin & Chu, 2018). Stata software version 11.2 (Stata Corp., College Station, TX) was used for the analyses. *p* < .050 was regarded as significant.

## 3 RESULTS

### 3.1 Search results

**FIGURE 1:**
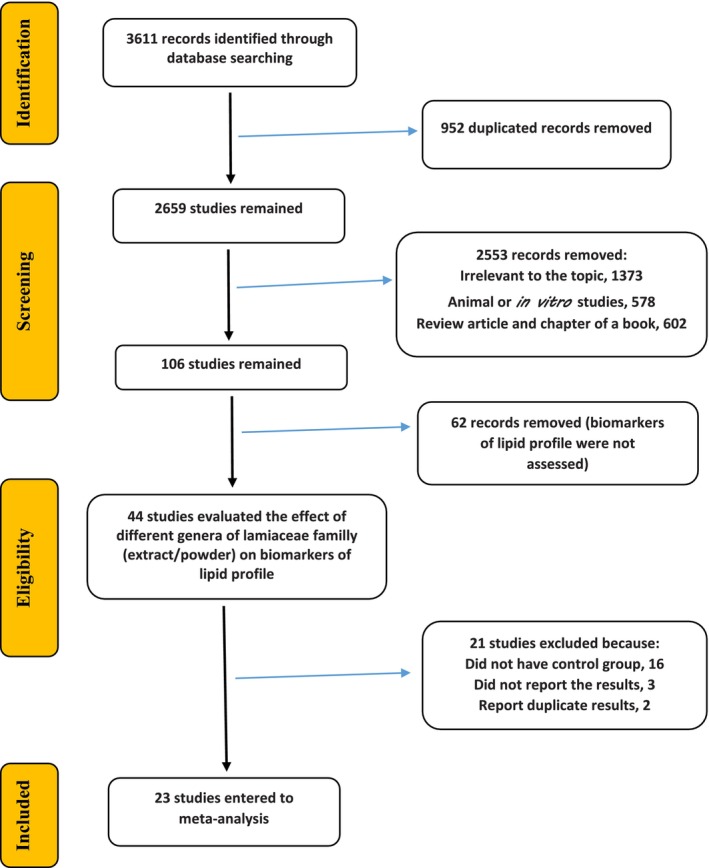
PRISMA flow diagram of study identification, inclusion, and exclusion.

According to Figure 1, a total of 3611 records were first obtained from electronic databases, and then 952 duplicate entries were excluded. Upon doing a more thorough evaluation, 2553 records were excluded due to their lack of relevance to the issue. In the end, 106 records were thoroughly assessed, and 23 studies (Abd El‐Ghany et al., 2014; Abolghasemi et al., 2020; Akbari et al., 2022; Alwosais et al., 2021; Arivuchudar et al., 2022; Asadi et al., 2019; Behradmanesh et al., 2013; Chusak et al., 2014; Jandaghi et al., 2016; S Kianbakht et al., 2011; Saeed Kianbakht et al., 2016; Loftus et al., 2015; Nieman et al., 2009; Nieman et al., 2012; Nikaein et al., 2017; Oliveira‐de‐Lira et al., 2018; Özdemir et al., 2008; Quaresma et al., 2023; Satapathy et al., 2016; Taleb et al., 2017; Tavares Toscano et al., 2014; Vosough‐Ghanbari et al., 2010; Vuksan et al., 2007) that fulfilled the specified criteria were chosen for this research.

### 3.2 Trial features

Table 1 presents the attributes of 23 qualified studies with a total of 1278 participants conducted in Australia, Brazil, Canada, Egypt, India, Iran, Kuwait, the USA, Thailand, and Turkey. The duration of the follow‐up ranged from 2 weeks to 3 months. The included studies recruited participants with various conditions, including hepatic failure (1 study), nonalcoholic fatty liver disease (1 study), overweight (2 studies), obese (2 studies), overweight/obese (5 studies), type 2 diabetes (4 studies), type 2 diabetes with dyslipidemia (4 studies), metabolic syndrome (1 study), and hyperlipidemia (3 studies).

**TABLE 1:** Specifications of randomized controlled trials included in the meta‐analyses.

Reference	Member of Lamiaceae family	Country	RCT design	No. of subjects	Health status	Intervention duration	Intervention group	Dose	Control group	Outcomes studied
Abd El‐Ghany et al. (2014)	*Rosmarinus officinalis*	Egypt	Parallel	I: 90 P: 30	Hepatic failure (post coma)	4 weeks	Leaf powder	5% of diet	Placebo	Cholesterol, Triglyceride, LDL, HDL
Abolghasemi et al. (2020)	*Zataria multiflora*	Iran	Parallel	I_1_: 32 I_2_: 31 P: 29	Overweight	12 weeks	Syrup	0.75, 1.5 g/day	Placebo (Oxymel)	Cholesterol, Triglyceride, LDL, HDL
Akbari et al. (2022)	*Rosmarinus officinalis*	Iran	Parallel	I: 57 P: 53	Nonalcoholic fatty liver disease	8 weeks	Leaf powder	4 g/day	Placebo (Starch)	Cholesterol, Triglyceride, LDL, HDL
Alwosais et al. (2021)	*Salvia hispanica*	Kuwait	Parallel	I: 20 P: 22	T2D	12 weeks	Seed	40 g/day	Did not consume seed	Cholesterol, Triglyceride, LDL, HDL
Arivuchudar et al. (2022)	*Ocimum basilicum*	India	Parallel	I_1_:10 I_2_:10 I_3_:10 P:10	T2D with dyslipidemia	45 days	Seed	20% raw, roasted or steamed seed/day	Did not consume seed	Cholesterol, Triglyceride, LDL, HDL
Asadi et al. (2019)	*Melissa officinalis*	Iran	Parallel	I: 31 P: 31	T2D	8 weeks	Hydroalcoholic extract	0.7 g/day	Placebo capsule (toasted flour)	Cholesterol, Triglyceride
Behradmanesh et al. (2013)	*Salvia officinalis*	Iran	Parallel	I: 40 P: 40	T2D	12 weeks	Extract	0.15 g/day	Placebo tablet	Cholesterol, Triglyceride, LDL, HDL
Chusak et al. (2014)	*Mesona chinensis*	Thailand	Cross‐over	I: 5 P:6	Overweight	2 weeks	Extract	0.5 g, 1 g/day	Basic meal	Triglyceride
Jandaghi et al. (2016)	*Melissa officinalis*	Iran	Parallel	I: 28 P: 30	Hyperlipidemic	8 weeks	Leaf powder	3 g/day	Placebo capsule (starch)	Cholesterol, Triglyceride, LDL, HDL
Kianbakht et al. (2011)	*Salvia officinalis*	Iran	Parallel	I: 34 P: 33	Hyperlipidemic	8 weeks	Leaf powder	1.5 g/day	Placebo capsule (toast powder)	Cholesterol, Triglyceride, LDL, HDL
Kianbakht et al. (2016)	*Salvia officinalis*	Iran	Parallel	I: 50 P: 50	T2D with dyslipidemia	8 weeks	Leaf powder	1.5 g/day	Placebo capsule (toast powder)	Cholesterol, Triglyceride
Loftus et al. (2015)	*Coleus forskohlii*	Australia	Parallel	I: 15 P: 15	Overweight and obese	12 weeks	Root extract	0.5 g/day	Placebo capsule (maltodextrin)	Cholesterol, Triglyceride, LDL, HDL
Nieman et al. (2009)	*Salvia hispanica*	USA	Parallel	I: 39 P: 37	Overweight and obese	12 weeks	Whole and milled seeds	50 g/day	Water	Cholesterol, Triglyceride, LDL, HDL
Nieman et al. (2012)	*Salvia hispanica*	USA	Parallel	I_1_: 16 I_2_: 14 P: 26	Overweight and obese	10 weeks	Whole and milled seeds	25 g/day	Placebo (poppy seed)	Cholesterol
Nikaein et al. (2017)	*Satureja hortensis*	Iran	Parallel	I: 24 P: 23	Metabolic syndrome	10 weeks	Essential oil	–	Placebo (capsule)	Cholesterol, Triglyceride, LDL, HDL
Oliveira‐de‐Lira et al. (2018)	*Salvia hispanica*	Brazil	Parallel	I: 19 P: 19	Obese	8 weeks	Oil	6 g/day	Placebo capsule (soybean oil)	Cholesterol, Triglyceride, LDL, HDL
Özdemir et al. (2008)	*Origanum onites*	Turkey	Parallel	I: 32 P: 16	Hyperlipidemic	12 weeks	Aqueous extract	6 mL/day	Water	Cholesterol, Triglyceride, LDL, HDL
Quaresma et al. (2023)	*Salvia hispanica*	Brazil	Parallel	I: 11 P: 9	Obese	90 days	Flour	30 g/day	Placebo (sachet)	Cholesterol, Triglyceride, LDL, HDL
Satapathy et al. (2016)	*Ocimum sanctum*	India	Parallel	I: 16 P: 14	Overweight and obese	8 weeks	Extract	0.5 g/day	Placebo (capsule)	Cholesterol, Triglyceride, LDL, HDL
Taleb et al. (2017)	*Thymus kotschyanus*	Iran	Parallel	I: 32 P: 32	T2D with dyslipidemia	12 weeks	Aqueous extract	20 g/day	Water	Cholesterol, Triglyceride, LDL, HDL
Tavares Toscano et al. (2014)	*Salvia hispanica*	Brazil	Parallel	I: 19 P: 7	Overweight and obese	12 weeks	Flour	35 g/day	Placebo	Cholesterol, Triglyceride, LDL, HDL
Vosough‐Ghanbari et al. (2010)	*Satureja khuzestanica*	Iran	Parallel	I: 11 P: 10	T2D with dyslipidemia	60 days	Leaf powder (tablet)	0.25 g/day	Placebo (tablet)	Cholesterol, Triglyceride, LDL, HDL
Vuksan et al. (2007)	*Salvia hispanica*	Canada	Cross‐over	I: 20 P: 20	T2D	12 weeks	Flour in bread	37 ± 4 g/day	Bread (wheat bran)	Cholesterol, Triglyceride, LDL, HDL

Abbreviations: HDL, high‐density lipoprotein; LDL, low‐density lipoprotein; RCT, randomized controlled trials; T2D, type 2 diabetes.

### 3.3 Evaluation of trial quality and synthesis of overall effects

Table 2 displays the outcomes of the quality assessment of trials based on the Cochrane recommendations. Despite the randomization of the studies included in the analysis, 2 studies did not provide enough information about how the random sequence was generated. Additionally, 13 studies did not adequately conceal the allocation process, 6 studies did not effectively blind the participants and personnel, 8 studies did not properly blind the assessment of outcomes, 1 study selectively reported outcomes, and 3 studies had other sources of bias. Furthermore, 3 studies were found to have a significant risk of bias in terms of allocation concealment, whereas 1 study had a risk of bias in terms of inadequate outcome data. No evidence of a significant risk of bias was identified for the other criteria assessed in the research. In all, 1 study was classified as “Low” quality, 3 studies were categorized as “Fair” quality, and 19 studies were categorized as “Good” quality.

**TABLE 2:** Risk of bias assessment of included randomized controlled trials according to the Cochrane guidelines.

Study	Random sequence generation	Allocation concealment	Blinding of participants and personnel	Blinding of outcome assessment	Incomplete outcome data	Selective outcome reporting	Other sources of bias^a^	Overall quality^b^
Abd El‐Ghany et al. (2014)	U	U	U	U	L	L	L	Fair
Abolghasemi et al. (2020)	L	U	L	L	L	L	L	Good
Akbari et al. (2022)	L	H	L	L	L	L	L	Good
Alwosais et al. (2021)	L	L	H	L	L	L	L	Good
Arivuchudar et al. (2022)	L	U	U	U	L	L	U	Fair
Asadi et al. (2019)	L	L	L	L	L	L	L	Good
Behradmanesh et al. (2013)	L	U	U	L	L	L	L	Good
Chusak et al. (2014)	U	U	U	U	H	L	U	Low
Jandaghi et al. (2016)	L	U	L	L	L	L	L	Good
Kianbakht et al. (2011)	L	L	L	L	L	L	L	Good
Kianbakht et al. (2016)	L	L	L	L	L	U	L	Good
Loftus et al. (2015)	L	L	L	L	L	L	L	Good
Nieman et al. (2009)	L	U	H	U	L	L	L	Good
Nieman et al. (2012)	L	U	L	L	L	L	L	Good
Nikaein et al. (2017)	L	L	L	L	L	L	L	Good
Oliveira‐de‐Lira et al. (2018)	L	H	L	U	L	L	L	Good
Özdemir et al. (2008)	L	U	U	U	L	L	L	Good
Quaresma et al. (2023)	L	U	H	L	L	L	L	Good
Satapathy et al. (2016)	L	H	H	U	L	L	U	Fair
Taleb et al. (2017)	L	U	U	U	L	L	L	Good
Tavares Toscano et al. (2014)	L	U	L	L	L	L	L	Good
Vosough‐Ghanbari et al. (2010)	L	U	L	L	L	L	L	Good
Vuksan et al. (2007)	L	L	H	L	L	L	L	Good

Abbreviations: H, high risk of bias; L, low risk of bias; U, unclear risk of bias.

^a^ Bias of study design, trial stopped early, extreme baseline imbalance, and fraudulent trial.

^b^ “Good” if at least 4 domains were low risk of bias, “Fair” if 3 domains were low risk of bias, “Low” if less than 3 domains were low risk of bias.

The evaluation of outcomes demonstrated a high level of meta‐evidence quality for cholesterol and triglyceride, suggesting that future research is unlikely to affect the confidence in the assessment of summary effects. The meta‐evidence quality for HDL and LDL was found to be moderate, suggesting that future research might potentially modify the level of confidence in the evaluation of summary effects (Table 3).

**TABLE 3:** Summary of findings with the NutriGrade scoring system.

Outcome	Effect size (95% CI)	No. of participants (trials)	Score	Outcome quality
Cholesterol	−11.88 mg/dL (−18.30 to −5.45)	1267 (25 trials)	8.3	High
Triglycerides	−12.04 mg/dL (−20.05 to −4.03)	1222 (27 trials)	8.3	High
HDL	1.95 mg/dL (−0.156 to 4.05)	1211 (25 trials)	7.3	Moderate
LDL	−8.43 mg/dL (−12.14 to −4.70)	1211 (25 trials)	7.8	Moderate

Abbreviations: CI, confidence interval; HDL, high‐density lipoprotein; LDL, low‐density lipoprotein.

### 3.4 Effect of Lamiaceae plants on blood lipid parameters

**FIGURE 2:**
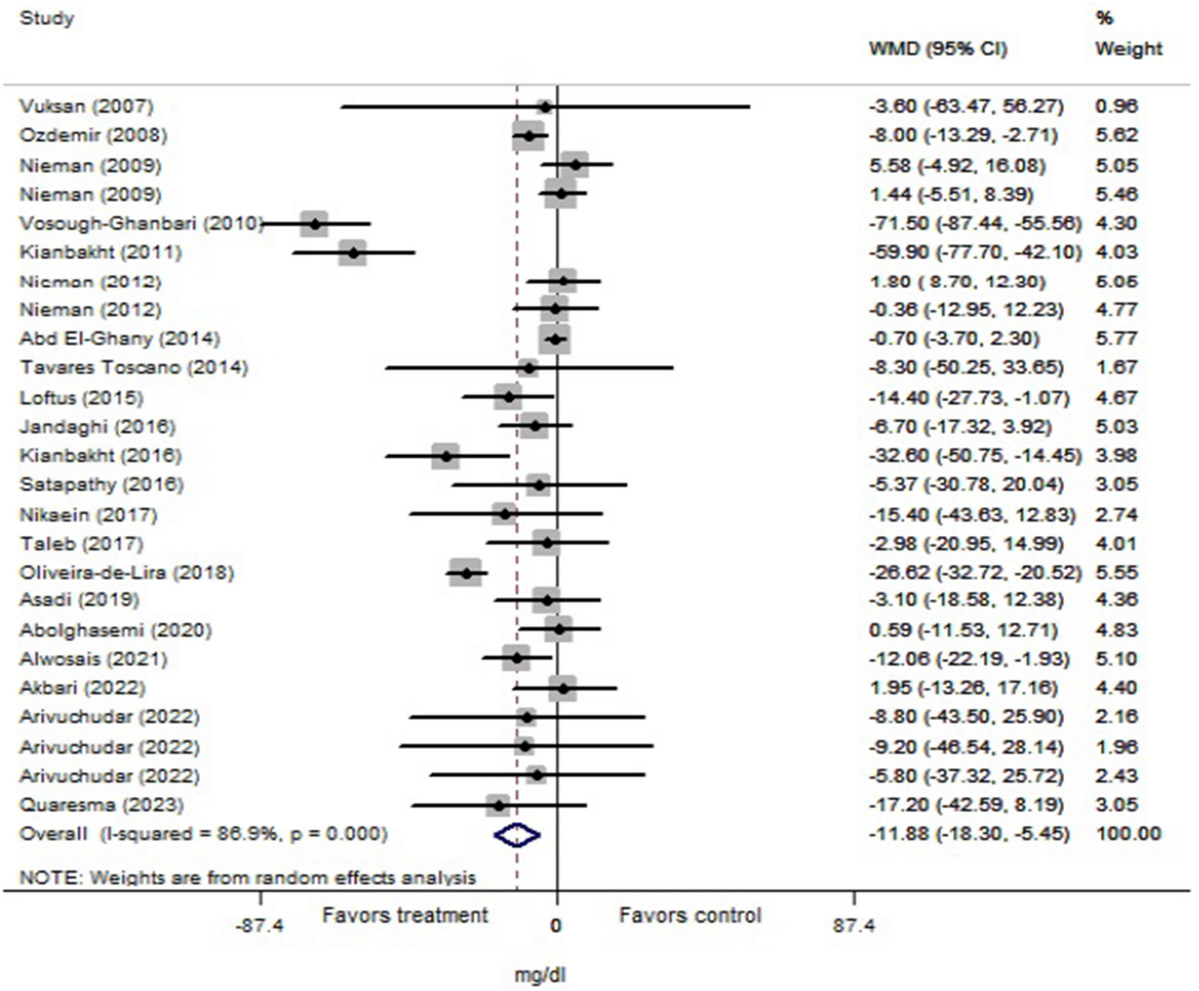
Forest plot of the effect of Lamiaceae plants on blood cholesterol level in patients with metabolic syndrome.

**FIGURE 3:**
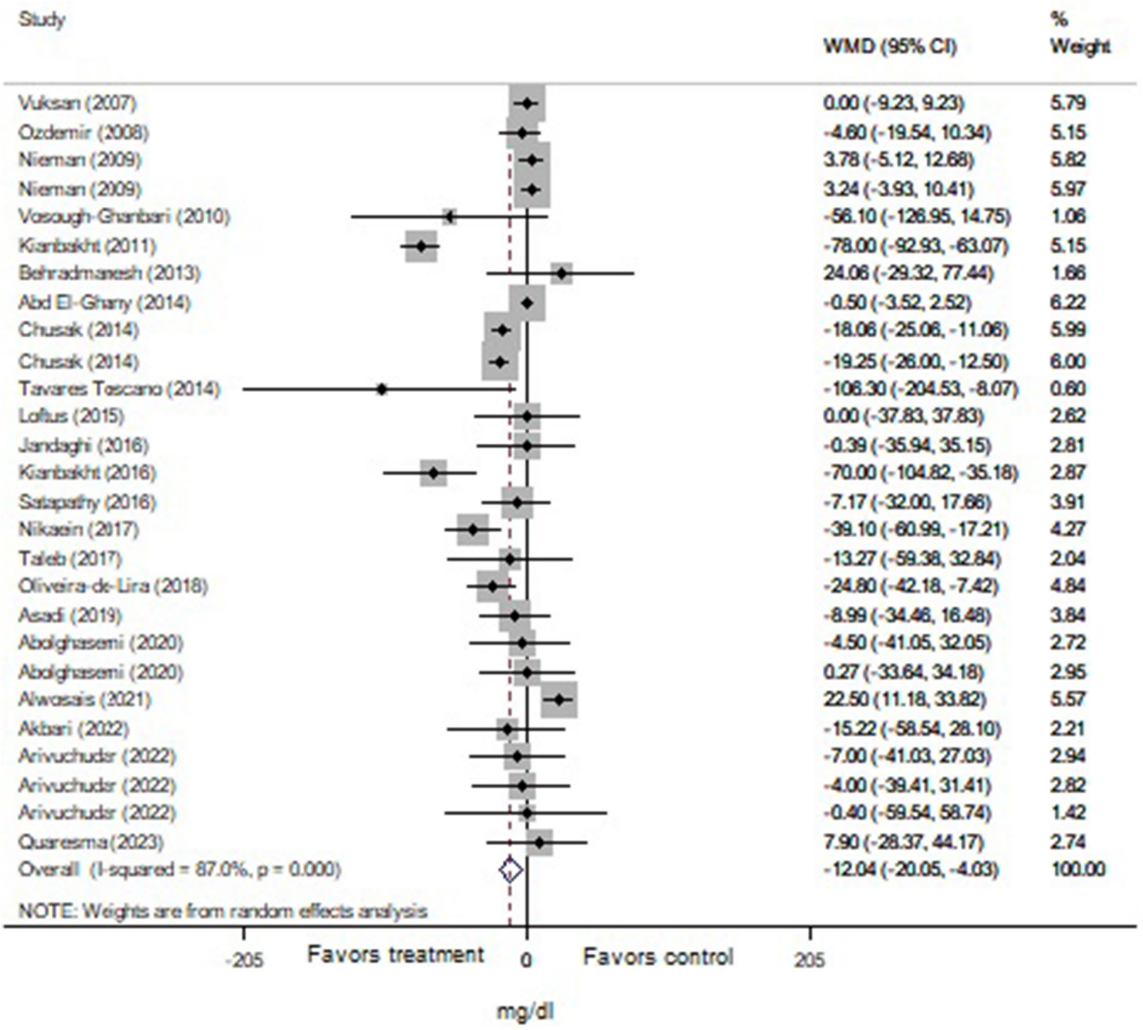
Forest plot of the effect of Lamiaceae plants on blood triglyceride level in patients with metabolic syndrome.

**FIGURE 4:**
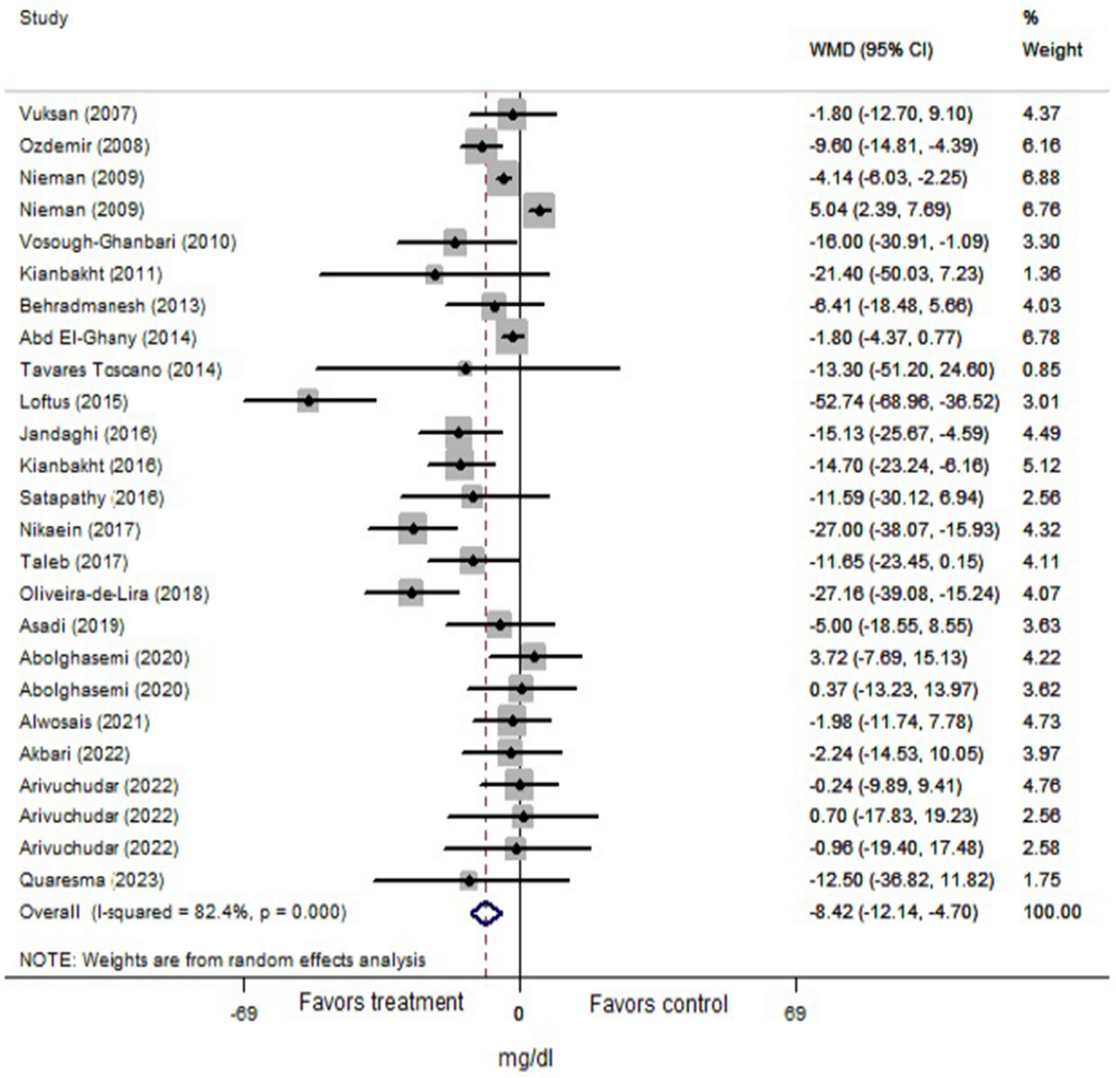
Forest plot of the effect of Lamiaceae plants on blood low‐density lipoprotein (LDL) level in patients with metabolic syndrome.

**FIGURE 5:**
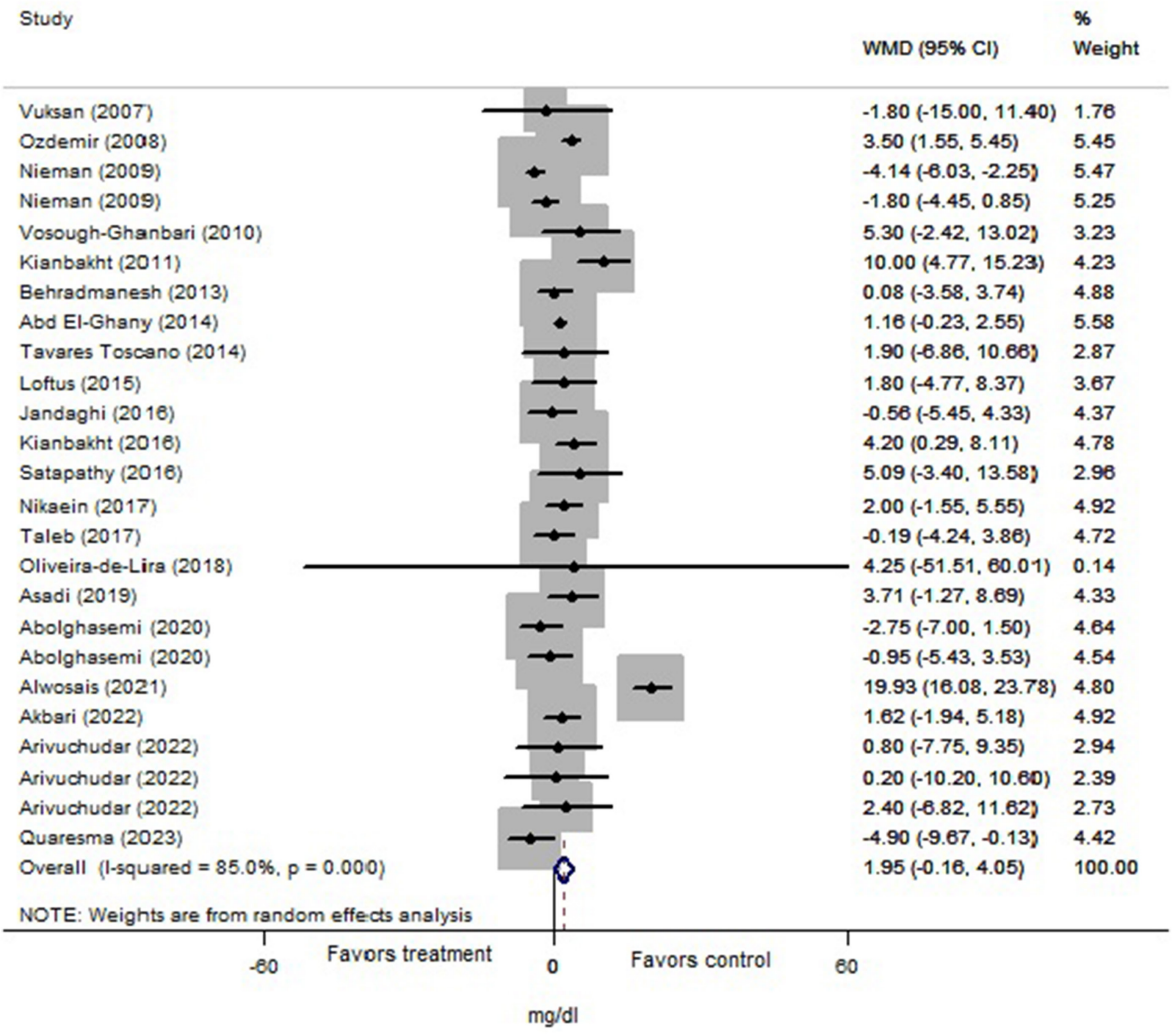
Forest plot of the effect of Lamiaceae plants on blood high‐density lipoprotein (HDL) level in patients with metabolic syndrome.

The total analysis was carried out on 25 trials from 22 articles (*n* = 1267 subjects) for cholesterol, 27 trials from 22 articles (*n* = 1222 subjects) for triglyceride, and 25 trials from 21 articles (*n* = 1211 subjects) for LDL and HDL that revealed significant reductions in levels of cholesterol (Figure 2: −11.88 mg/dL, 95% CI: −18.30 to −5.45; *p* < .001), triglyceride (Figure 3: −12.04 mg/dL, 95% CI: −20.05 to −4.03; *p* = .003), and LDL (Figure 4: −8.43 mg/dL, 95% CI: −12.14 to −4.70; *p* < .001) following Lamiaceae plant intake; but the elevation of HDL level (Figure 5: 1.95 mg/dL, 95% CI: −0.156 to 4.05; *p* = .069) was not significant. However, inter‐trial heterogeneity was considerable for cholesterol, triglyceride, LDL, and HDL based on the I2 index values (cholesterol: 86.9%; triglyceride: 87.0%; LDL: 82.4%; HDL: 85.0%) and Cochrane Q test (*p* < .001 for all lipid parameters). The sensitivity analyses for the aforementioned lipid parameters failed to identify any study that contributed to the observed heterogeneity.

### 3.5 Analyses of subgroups

The results of subgroup analyses, including intervention duration, metabolic status, and Lamiaceae genera, are presented in Table 4. The study revealed that both short‐term (≤8 weeks) and long‐term (>8 weeks) consumption of Lamiaceae plants resulted in a reduction in LDL levels in the blood (*p* < .05). However, only the short‐term consumption of these plants resulted in reduced triglyceride and cholesterol levels, along with elevated HDL levels (*p* < .001).

**TABLE 4:** Results of the effect of Lamiaceae plants on biomarkers of lipid profile based on subgroup analyses.

Outcome	Variable	No. of trials	Effect size as mg/dl (95% CI)	*p*‐value	I^2^ (%)	Q‐statistics (P)
**Cholesterol**	Intervention duration					
	≤8 weeks	12	−19.77 (−32.80, −6.73)	.003	93.2	< .001
	>8 weeks	13	−3.84 (−7.80, 0.124)	.058	26.3	.179
	Metabolic status					
	Hepatic disorders	3	−0.76 (−3.68, 2.17)	.611	0.0	.561
	Overweight/obese	10	−5.82 (−15.41, 3.78)	.235	84.3	< .001
	T2D/T2D with dyslipidemia	9	−18.49 (−36.35, −0.63)	.042	85.2	< .001
	Hyperlipidemia	3	−23.22 (−47.96, 0.23)	.052	93.5	< .001
	Genera of Lamiaceae					
	*Rosmarinus*	2	−0.60 (−3.54, 2.34)	.689	0.0	.738
	*Zataria/Thymus*	2	−0.53 (−10.58, 9.52)	.918	0.0	.747
	*Salvia*	11	−13.86 (−25.27, −2.45)	.017	88.9	< .001
	*Ocimum*	4	−6.84 (−22.45, 8.77)	.391	0.0	.998
	*Melisa*	2	−5.55 (−14.31, 3.21)	.214	39.6	.707
	*Mesona*	‐	–	–	–	–
	*Coleus*	1	−14.40 (−27.73, −1.07)	.034	–	–
	*Satureja*	2	−44.71 (−99.63, 10.21)	.111	91.3	.001
	*Origanum*	1	−8.00 (−13.29, −2.71)	.003	–	–
**Triglyceride**	Intervention duration					
	≤8 weeks	14	−21.84 (−34.49, −9.20)	< .001	91.0	< .001
	>8 weeks	13	−0.12 (−8.68, 8.45)	.979	63.8	.001
	Metabolic status					
	Hepatic disorders	3	−17.19 (−46.38, 12.00)	.248	83.5	.002
	Overweight/obese	11	−8.71 (−18.00, 0.57)	.066	76.6	< .001
	T2D/T2D with dyslipidemia	10	−7.34 (−23.77, 9.10)	.382	73.3	< .001
	Hyperlipidemia	3	−28.64 (−84.46, 27.19)	.315	96.1	< .001
	Genera of Lamiaceae					
	*Rosmarinus*	2	−0.57 (−3.59, 2.4)	.711	0.0	.506
	*Zataria*/*Thymus*	3	−4.49 (−26.37, 17.39)	.688	0.0	.898
	*Salvia*	10	−15.61 (−34.66, 3.43)	.108	93.9	.000
	*Ocimum*	4	−5.88 (−22.62, 10 .86)	.491	0.0	.996
	*Melisa*	2	−6.07 (−26.78, 14.3)	.565	0.0	.700
	*Mesona*	2	−18.68 (−23.53, −13.81)	< .001	0.0	.810
	*Coleus*	1	0.00 (−37.83, 37.83)	1.000	–	–
	*Satureja*	2	−40.58 (−61.50, −19.67)	< .001	0.0	.653
	*Origanum*	1	−4.60 (−19.54, 10.34)	.546	–	–
**HDL**	Intervention duration					
	≤8 weeks	12	2.70 (1.03, 4.36)	< .001	24.4	.204
	>8 weeks	13	1.07 (−2.40, 4.54)	.546	91.5	< .001
	Metabolic status					
	Hepatic disorders	3	1.31 (−0.10, 2.53)	.063	0.0	.896
	Overweight/obese	9	−2.30 (−3.95, 0.25)	.056	21.8	.249
	T2D/T2D with dyslipidemia	10	3.88 (−1.24, 9.00)	.137	87.9	< .001
	Hyperlipidemia	3	4.15 (−0.58, 8.87)	.085	76.5	.014
	Genera of Lamiaceae					
	*Rosmarinus*	2	1.22 (−0.07, 2.52)	.065	0.0	.813
	*Zataria*/*Thymus*	3	−1.27 (−3.73, 1.18)	.309	0.0	.684
	*Salvia*	10	2.77 (−2.70, 8.24)	.321	93.8	< .001
	*Ocimum*	4	2.30 (−2.24, 6.84)	.321	0.0	.876
	*Melisa*	2	1.55 (−2.64, 5.73)	.469	30.4	.231
	*Mesona*	–	–	–	–	–
	*Coleus*	1	1.80 (−4.77, 8.37)	.591	–	–
	*Satureja*	2	2.58 (−0.65, 5.81)	.118	0.0	.447
	*Origanum*	1	3.50 (1.55, 5.45)	< .001	–	–
**LDL**	Intervention duration					
	≤8 weeks	12	−9.00 (−14.54, −3.46)	.001	66.7	.001
	>8 weeks	13	−8.33 (−13.99, −2.67)	.004	88.2	< .001
	Metabolic status					
	Hepatic disorders	3	−9.93 (−25.22, 5.35)	.203	89.4	< .001
	Overweight/obese	9	−10.30 (−18.14, −2.45)	.010	91.1	< .001
	T2D/T2D with dyslipidemia	10	−6.33 (−10.26, −2.40)	.002	9.7	.354
	Hyperlipidemia	3	−10.96 (−15.57, −6.36)	< .001	0.0	.503
	Genera of Lamiaceae					
	*Rosmarinus*	2	−1.82 (−4.33, 0.70)	.156	0.0	.945
	*Zataria*/*Thymus*	3	−2.59 (−12.09, 6.91)	.593	44.8	.163
	*Salvia*	10	−7.16 (−13.05, −1.27)	.017	85.2	< .001
	*Ocimum*	4	−1.90 (−9.06, 5.26)	.602	0.0	.741
	*Melisa*	2	−11.00 (−20.75, −1.24)	.027	25.8	.247
	*Mesona*	–	–	–	–	–
	*Coleus*	1	−52.74 (−68.96, −36.52)	< .001	–	–
	*Satureja*	2	−22.68 (−33.21, −12.15)	< .001	25.8	.246
	*Origanum*	1	−9.60 (−14.81, −4.39)	< .001	–	–

*Note:* The *p* < .05 was statistically considered significant.

Abbreviations: HDL, high‐density lipoprotein; LDL, low‐density lipoprotein; T2D, type 2 diabetes.

The meta‐analysis of metabolic status subgroups revealed that Lamiaceae plants had a significant effect on reducing LDL and cholesterol levels in diabetic patients, as well as LDL levels in hyperlipidemic and overweight/obese individuals (*p* < .05).

Upon evaluating the Lamiaceae family, it was found that only the genera *Salvia*, *Coleus*, and *Origanum* could decrease blood cholesterol levels (*p* < .034). Similarly, the genera *Salvia*, *Melisa*, *Origanum*, *Coleus*, and *Satureja* were found to decrease blood LDL levels (*p* ≤ .027). The genera *Satureja* and *Menosa* were the only ones that reduced blood triglyceride levels (*p* < .001). Lastly, the *Origanum* genus was the only one that increased blood HDL levels (*p* < .001). The blood lipid profile did not show substantial changes in other genera, including *Rosmarinus*, *Zataria*/*Thymus*, and *Ocimum*.

## 4 DISCUSSION

Lipids are a major category of biomolecules that serve several activities, including being components of membranes, molecules engaged in signaling, and storage of energy. They also play a crucial role in essential cellular processes. Lipid metabolism is tightly regulated to maintain homeostasis, and it demonstrates intricate spatial and dynamic complexity at several levels. Disruptions in the lipid profile can be linked to diseases. Therefore, understanding the precise deviations in lipid parameters for each disease holds great potential for diagnosing and predicting the course of the disease. Additionally, this knowledge is crucial for uncovering the underlying pathophysiological mechanisms of diseases and developing novel and more effective therapeutic strategies (Alves et al., 2021). Research has shown that when lipid profile is impaired in metabolic disorders such as hyperlipidemia, diabetes, overweight/obesity, and fatty liver disease, the risk of life‐threatening conditions like cardiovascular and neurodegenerative diseases increases. Therefore, it is crucial to improve lipid profile control in individuals (Alves et al., 2021; Arsenault et al., 2011).

Previous studies reviewed the potential of Lamiaceae herbs to alleviate overweight, obesity, and fatty liver (Diab et al., 2022; Pavlos & Nikiforou, 2022). This meta‐analysis further validated the overall advantages of these plants in enhancing lipid profile. The primary contributors to the enhancement of lipid profile are the flavonoids and phenolic chemicals found in these plants. These chemicals have the ability to diminish the desire to eat and enhance the feeling of satiety. Flavonoids primarily enhance the levels of AMP‐dependent protein kinase (AMPK) and sirtuin 1 (SIRT1), which are the key factors accountable for reducing lipogenesis, increasing lipolysis, and promoting the expression of proliferator‐activated receptor gamma coactivator 1‐α (PGC‐1α). The AMPK/PGC1α signaling pathway is the main regulator of adipose tissue and thermogenesis (Bhardwaj et al., 2021; Chakrabarti et al., 2011; Fulco & Sartorelli, 2008). Promoting thermogenesis and increasing energy expenditure is a crucial method for reducing obesity (Bhardwaj et al., 2021). AMPK also promotes the breakdown of fatty acids and decreases the production of cholesterol by disrupting the activity of fatty acid synthase and 3‐hydroxy‐3‐methyl‐glutaryl‐CoA reductase (Bhardwaj et al., 2021).

In the present review, the meta‐analysis of data indicated that short‐ and long‐term intakes of Lamiaceae plants could lower blood LDL levels. Elevated levels of LDL are widely acknowledged as significant risk factors for the occurrence of atherosclerosis, increasing the likelihood of catastrophic cardiovascular events such as heart attacks and strokes. LDL also serves as a regulator of vascular function, exhibiting dynamic properties involving vasoconstriction, cell growth stimulation, promotion of inflammation, thrombus formation, and recruitment of immune cells. LDL enhances noradrenaline‐induced vasoconstriction in the peripheral vasculature, as well as in the coronary, cerebral, and renal vascular beds. In addition, there is a decrease in the ability of the endothelium to dilate blood vessels in response to acetylcholine. LDL additionally exerts its vasoconstrictive impact by suppressing the activity of endothelial nitric oxide synthase (Rosendorff, 2002). Nevertheless, the role of Lamiaceae plants in reducing LDL levels may be beneficial in mitigating cardiovascular events. Our investigation revealed that these plants had a short‐term effect of reducing cholesterol and triglyceride levels while increasing HDL levels. However, this particular trait may be considered a drawback. Nevertheless, this serves as evidence that the length of intervention with Lamiaceae plants might have a significant role in their effectiveness on lipid biomarkers.

According to the findings of our meta‐analysis, patients with type 2 diabetes received the greatest benefits from consuming Lamiaceae. As a result of the fact that these plants improved two lipid parameters (LDL and cholesterol) in diabetic patients, whereas in other categories of patients with metabolic syndrome, none or only one parameter was significantly altered. The criticality of this improvement is underscored by the fact that dyslipidemia, which arises from the coexistence of hyperglycemia and insulin resistance in diabetic patients, remains the primary cause of mortality and disability among these patients (Low Wang et al., 2016).

In subgroup meta‐analysis of Lamiaceae genera, it was revealed that *Origanum* genus improves more lipid parameters (HDL, LDL, and cholesterol) in patients with metabolic syndrome. Carvacrol and thymol are main constituents of this genus (Marrelli et al., 2018) and their antihyperlipidemic effects are well known in previous studies (Aristatile et al., 2009; Salari et al., 2021; Saravanan & Pari, 2015).

There are limitations to this study. (1) The chosen trials utilized several species from the Lamiaceae family, each with distinct compositions and active chemicals. (2) The participants in the chosen trials had different metabolic issues. (3) Out of the 23 selected studies, two studies (Chusak et al., 2014; Vuksan et al., 2007) had a crossover design, which differed from the others. Additionally, one study (Chusak et al., 2014) did not include a washout period during the trial. (4) The dosage of many studied plants was not clear (Abd El‐Ghany et al., 2014; Arivuchudar et al., 2022; Nikaein et al., 2017; Özdemir et al., 2008), and therefore an independent subgroup as “intervention dosage” could not be identified.

The strength of this survey is that the high quality of meta‐evidence ensures the reliability of the results regarding overall effects on cholesterol, triglycerides, LDL, and HDL.

## 5 CONCLUSIONS

This meta‐analysis study determined that consumption of Lamiaceae plants reduced the levels of cholesterol, triglyceride, and LDL, while changes in the blood HDL levels were not statistically significant. Subgroup studies revealed that consuming Lamiaceae plants improves all lipid parameters for a short duration while in a longer duration, only LDL level is improved. Type 2 diabetic individuals had more advantages from consuming Lamiaceae as it led to a notable decrease in LDL and cholesterol levels. The genera *Satureja* and *Coleus* had the most significant effects in decreasing triglyceride and LDL/cholesterol levels, respectively, and *Origanum* was the only genus within the Lamiaceae family that significantly improved three lipid parameters (cholesterol, LDL, and HDL).

## AUTHOR CONTRIBUTIONS

Hossein Hassanpour: Conceptualization (equal); investigation (equal); methodology (equal); software (equal); writing—original draft (equal); writing—review and editing (equal). Aziz A. Fallah: Conceptualization (equal); data curation (equal); formal analysis (equal); methodology (equal); software (equal); writing—original draft (equal); writing—review and editing (equal). Tina Jafari: Formal analysis (equal); methodology (equal); software (equal); writing—original draft (equal); writing—review and editing (equal). Marzieh Mojtahed: Data curation (equal); software (equal); writing—review and editing (equal).

## CONFLICT OF INTEREST STATEMENT

The authors declare no conflicts of interest.

## ETHICS STATEMENT

This study does not involve any human or animal testing.

## Data Availability

Data sharing is not applicable to this article as no new data were created or analyzed in this study.
